# Editorial: Frontiers in the acquisition of literacy

**DOI:** 10.3389/fpsyg.2015.01019

**Published:** 2015-07-17

**Authors:** Claire M. Fletcher-Flinn

**Affiliations:** School of Psychology, The University of AucklandAuckland, New Zealand

**Keywords:** reading acquisition theory, alphabetism, predictors of reading, spelling, reading intervention and methodology, reading comprehension

Reading and writing are fundamental to full participation in our societies, yet how children acquire such a large system of interconnected representations of print words, their meanings, and phonology in the brain remains unclear. As the teaching of literacy takes up a large proportion of classroom time in the early years, increasing knowledge about children's learning processes should result in better approaches to the teaching of reading and spelling. These insights would be particularly useful from a clinical perspective for the treatment of developmental disabilities, such as dyslexia and dysgraphia.

Important questions need to be addressed, and given the many different influences and overlapping processes on literacy learning, the answers are not straightforward. What are the learning processes? Are they the same across different orthographies, or do different orthographies require different skills and learning processes? What is the relationship between reading and spelling? How do they interact and augment each other? What is the effect of different teaching approaches on children's emerging reading system? Do early reading comprehension problems disappear over time? What are the predictors of children's reading attainment?

This E-Book responds to some of these general questions in the form of original research and opinion articles. Taken together the articles contribute new perspectives and challenges to current reading acquisition theories, and present new research on early reading skills, reading instruction, and spelling.

Thompson (2014a) claims that most reading acquisition theories are limited by their specification of letter-sound requirements to a particular class of teaching approaches. Acknowledging such limitations is an important step in the development of reading acquisition theories that are potentially more useful. Fletcher-Flinn (2014) merges ideas from dynamic systems theory with developmental data from a precocious reader. Reference is made to Knowledge Systems theory, which offers a more varied range of theoretical applicability.

Share (2014) asserts that there is a general belief in the superiority of alphabetic writing systems that has hindered progress in the development of a universal model of learning to read. Some counterevidence is presented, and in the context of a more general question on “optimality,” Share proposes a universal model of reading based on a broader novice-to-expert dualism. Nag (2014) maintains that specification of the learning mechanisms involved in reading akshara units, the symbols used in many writing systems (alphasyllabaries) of Southern Asia, present a challenge to alphabet-based theories of reading acquisition. The akshara units, unlike alphabet letters, map onto multiple sublexical levels of phonology determined by context. In order to ensure the development of an inclusive reading science, and a more comprehensive and universal theory of literacy learning, Nag argues that consideration of these orthographic-specific features of reading are needed. At the same time, it is possible that some general cognitive features of information processing, as they relate to reading acquisition, may be orthography-independent. Colé et al. (2014) provides evidence that early progress in young French children in both word reading and reading comprehension was related to cognitive flexibility in the coordination of orthographic, phonological, and semantic information.

How early, and in what form does SES as a distal predictor of reading achievement manifest itself? Robins et al. (2014) found that lower SES parents of preschoolers asked fewer questions about letters, and focused more on memorizing sequences of the alphabet than higher SES parents. The persistence of a conversational focus on letters within the child's name also differentiated the groups. These differences could put lower SES children at a disadvantage when entering school, resulting in poor rates of literacy. Tse and Nicholson (2014) addressed the performance gap of low SES children in New Zealand schools with an intervention comparing three teaching approaches: Big Book (shared reading), explicit instruction in phoneme awareness and phonics, and a combined approach. The latter produced better results on a range of literacy measures compared with the combined averaged scores of the other two groups. Thompson (2014b) took the opportunity to comment on ambiguities that are often unrecognized but affect the validity of such intervention research, and Nicholson and Tse (2015) provide a rebuttal. These discussions are thoughtful contributions to methodological issues in intervention research.

How do fluent readers distinguish between words that look similar but whose meaning differ? Using masked form priming, Bhide et al. (2014) found no evidence that increases in print vocabulary size predicted precise orthographic representations, and suggested spelling skill might be more important. Ouellette and Tims (2014) examined whether this “spelling advantage” might be due to the motoric component of writing. There was no effect of modality (printing or typing) for Grade two children, suggesting that stored orthographic detail is independent of input. Of interest, pre-existing keyboard skills affected learning.

With regard to literacy impairments, Critten et al. (2014) found no difference for the spelling of words with inflectional morphemes by children with specific language impairment (SLI) and spelling-matched controls. However, the SLI group was less accurate when spelling words with derivational morphemes. The authors conclude that this indicates a specific impairment when making orthographic and phonological shifts from base words. This outcome has useful teaching applications for SLI children. Ricketts et al. (2014) showed that children identified at 9 years with poor reading comprehension had lower educational achievement at 11 and 16 years than a reading (decoding and comprehension), and non-verbal reasoning matched group, and were below national performance norms. They point out that these children are at risk from an early age of a compromised future with regard to further training and employment.

This E-Book contains an excellent collection of cutting edge scientific research and opinions at the frontiers of literacy acquisition. The reader will find new perspectives and questions derived from the reported findings, and these can serve as a springboard for new research in this field.

## Conflict of interest statement

The author declares that the research was conducted in the absence of any commercial or financial relationships that could be construed as a potential conflict of interest.
